# Quality of discharge letters concerning readmissions to mental healthcare: case-control study

**DOI:** 10.1192/bjo.2025.56

**Published:** 2025-05-23

**Authors:** Miriam Hartveit, Harald Bjarne Hellesen, Jostein Helgeland, Olav Thorsen, Jörg Assmus, Eva Biringer

**Affiliations:** Department of Research and Innovation, Fonna Hospital Trust, Valen, Norway; Department of Global Public Health and Primary Care, University of Bergen, Bergen, Norway; Suldal General Practitioners Center, Suldal Municipality, Suldal, Norway; Haugesund Health Care Office, Haugesund Municipality, Haugesund, Norway; Stavanger University Hospital, Stavanger, Norway; Centre for Clinical Research, Bergen Hospital Trust, Bergen, Norway; Department of Research and Innovation, Fonna Hospital Trust, Stord, Norway

**Keywords:** Patient discharge, patient readmission, mental health, patient handoff

## Abstract

We investigated the quality of 100 discharge letters from mental health specialists to better understand the role of cross-sectoral communication in patients’ readmissions or other unplanned recontacts with mental health specialist care. Using a case-control design, we compared 50 letters for patients readmitted or for whom a formal request for additional specialist mental healthcare was made by the patient’s GP (recontact) within 30 days of discharge, and 50 letters for those without readmission or recontact. The 26-item checklist Quality of Discharge letters – Mental Health was used to assess discharge letter quality. No significant differences in total checklist scores were found, suggesting that discharge letter quality does not influence the rates of readmission or unplanned recontact in mental healthcare.

Readmission rates to mental healthcare services are high,^1–4^ with incomplete follow-ups and weak responsiveness of community services linked to rehospitalisation.^5^ Discharge planning and coordination reduce the risk of readmission,^6^ and systems aimed at standardising and ensuring the availability of discharge summaries have been recommended.^7^ A discharge summary or letter is a mandatory written report sent from specialist care to the GP on discharge about the patient’s diagnosis, received specialist care and suggestions for further follow-up, and their potential impact on patient risks and outcomes has been emphasised.^8,9^ However, a case-control study by Hansen et al, which included mainly patients with medical conditions, found no association between documentation of mandatory elements in discharge letters and the likelihood of 30-day hospital readmission.^10^ We did not find similar studies in the mental healthcare literature. Furthermore, no research has examined whether incomplete discharge letters contribute to unplanned recontacts – that is, new referrals or formal requests for additional specialist assessments initiated by primary care physicians.

## Aim

This study aimed to investigate whether the quality of discharge letters for mental health patients with unplanned recontacts (readmissions or other recontacts) within 30 days of discharge was lower than that for mental health patients without unplanned recontacts.

## Method

Using a retrospective case-control design, we compared the discharge letters of patients with and without recontact with specialist mental health services within 30 days of discharge. We used a retrospective case-control design, because this is recommended for investigation of phenomena with little existing evidence in naturalistic settings. From 1 October 2019 and approximately 6 months backwards in time, 100 discharge letters were retrospectively retrieved from the electronic patient records of the mental health services at Fonna Hospital Trust in western Norway. Discharge letters were consecutively sampled by administrative staff until 50 letters from patients with recontact (‘cases’) and 50 from patients without recontact (‘controls’) were obtained. The recontact group included 40 discharge letters from readmitted patients and 10 from patients whose GP had initiated recontact for other reasons. The sample size was determined *a priori*, based on a power analysis estimate that a sample of 31 discharge letters was required to detect significant group differences (two-tailed bivariate correlation; power 0.8, significance level 0.05, correlation 0.5). For reasons of confidentiality, patient identification details were excluded by the administrative staff.

**Fig. 1:**
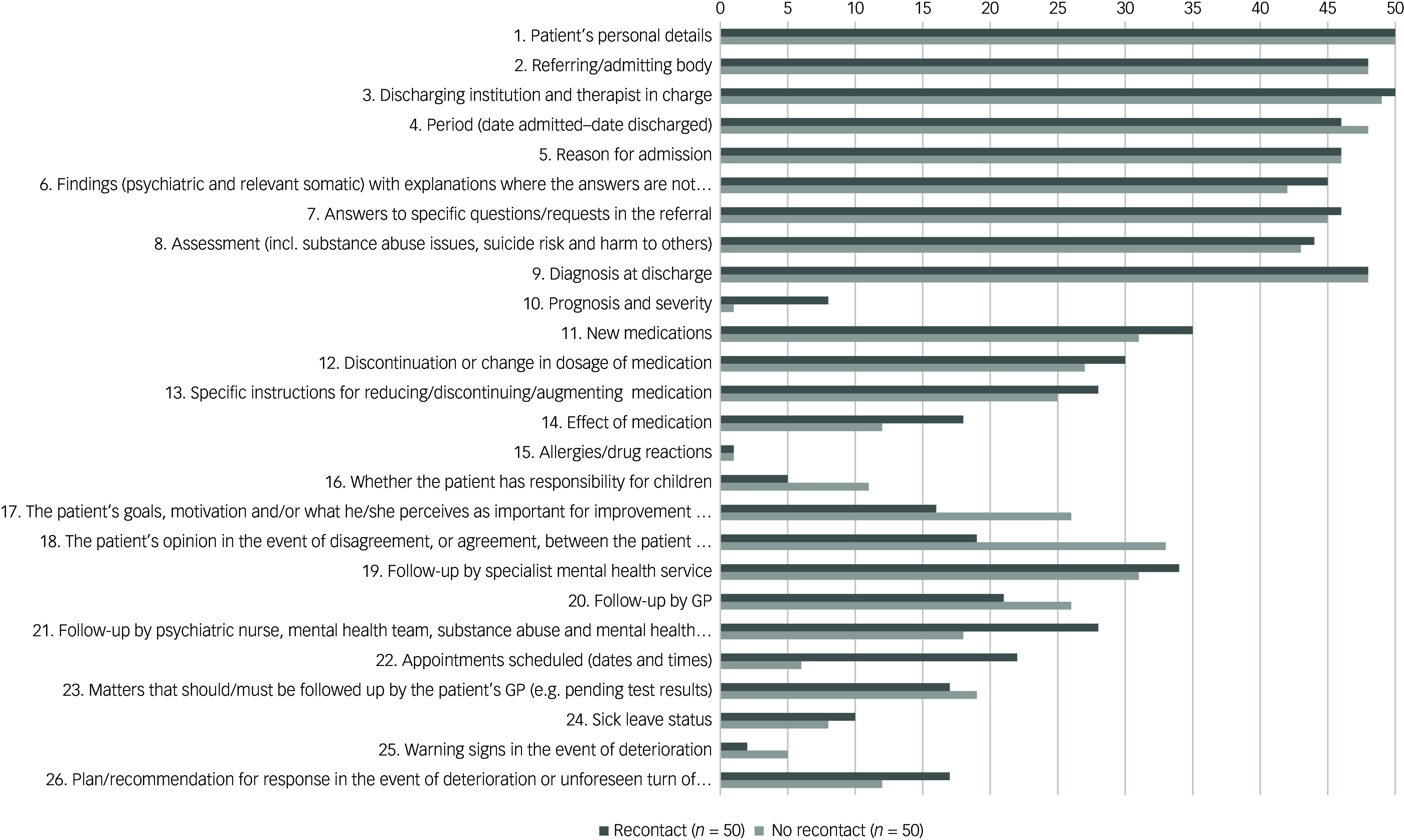
Frequencies of discharge letters providing specific information items as assessed by the QDis-MH checklist. Discharge letters from the recontact group (*n* = 50) versus the no recontact group (*n* = 50). GP, general practitioner.

The 26-item Quality of Discharge letters – Mental Health (QDis-MH) checklist was used to assess the quality of discharge letters according to whether specific items of information were present (1) or absent (0) in each discharge letter^11^ (Fig. 1). To optimise the validity of the scores, two independent raters (MH and EB) scored the discharge letters using the QDis-MH checklist, with minor discrepancies resolved through consensus. Before the averaged QDis-MH total scale was calculated, item 1 about patients’ personal details was scored 1 in all discharge letters. That was done because this information is automatically included in all discharge letters in the electronic patient record system in this setting. The QDis-MH total scale was then calculated as described by Biringer et al.^11^ Group differences in QDis-MH total scores were analysed using Student’s *t*-test for independent samples, while item-level differences were assessed using *χ* ^2^-tests. The significance level was set at 0.05.

### Ethics statement

Informed consent was not obtained from patients, because discharge letters were anonymised by administrative staff before delivere to the researchers. The Norwegian Social Science Data Service (reference no. 53392) and the Regional Committees for Medical and Health Research Ethics in Norway approved the study, including the scoring of anonymised discharge letters without patient consent (REK Nord reference no. 2019/1147). ChatGPT-4.o (OpenAI) was used to refine the language and grammar in some sentences. The final content was reviewed and approved by the authors.

## Results

Descriptive statistics of the patients represented by the 100 discharge letters are shown in Supplementary Table 1 available at https://doi.org/10.1192/bjo.2025.56.

The 100 discharge letters contained a mean of 14.5 (s.d. = 2.7, range 7–20) of the QDis-MH checklist information items. There were no significant differences in the number of information items reported between the groups recontact (mean (50) = 14.7 (s.d. = 2.9, range 9–20)) and no recontact (mean (50) = 14.3 (s.d. = 2.6, range 7–20); *P* = 0.987, Student’s *t*-test).

Figure 1 illustrates the frequency of discharge letters containing specific information items, as assessed by the QDis-MH checklist. Significantly fewer discharge letters in the recontact group included information on item 17: ‘The patient’s goals, motivation and/or what he/she perceives as important for improvement and development’ (*χ* ^2^ (1, *N* = 93) = 4.74, *P* ≤ 0.05) and item 18: ‘The patient’s opinion in the event of disagreement, or agreement, between the patient and the clinical treatment team’ (*χ* ^2^ (1, *N* = 91) = 9.53, *P* ≤ 0.05). Information on item 22 regarding future scheduled appointments was significantly more frequent in the discharge letters of the recontact group compared with the no-recontact group (*χ* ^2^ (1, *N* = 95) = 12.49, *P* ≤ 0.001). All other group comparisons at the item level were not significant (*P* > 0.05).

## Discussion

The study suggests that the overall quality of the discharge letter does not influence patients’ 30-day readmission or recontact rates between GPs and mental health specialist care, which is consistent with the findings of Hansen et al.^10^ Our findings suggest that rates of readmission or recontact with mental health specialist services are more likely to be determined by factors other than the quality of discharge letters – for example, illness severity, functioning level or contextual factors, such as the availability of supportive community services. In our sample, recontact patients more frequently suffered from severe mental illness or personality disorders, and were more likely to have used specialist services in the previous 5 years than no-recontact patients. This is consistent with existing research.^13–15^ These group differences may explain why information about future appointments was more frequently included in discharge letters in the recontact group in our study, because such follow-up arrangements are more relevant for patients with severe conditions and low functioning. Previous research has emphasised patient involvement in planning their discharges and post-discharge follow-up.^12^ Patient participation is particularly important for those with frequent admissions or unplanned recontacts, especially if they are more severely ill than patients without recontacts, as was the case in our study. We were therefore surprised to find that recontact group letters contained less information on patients’ personal goals, motivations and opinions about their care compared with no-recontact group letters.

### Limitations

This study did not match recontact and no-recontact groups for illness severity, or for other factors that may moderate the associations between the quality of discharge letter and unplanned readmission or recontact rates. Another potential limitation is the suboptimal reliability of the checklist used. Our sample reflects mental health patients in Norway’s public specialist care facilities and, while the findings are perhaps applicable to similar settings in Western countries, their generalisability to other healthcare contexts remains uncertain.

Our study found no differences in the overall quality of discharge letters between mental healthcare patients with and without unplanned recontacts (readmissions or other recontacts) within 30 days, suggesting that discharge letter quality may not influence unplanned readmission or recontact rates in mental healthcare.

## Supporting information

Hartveit et al. supplementary material Hartveit et al. supplementary material

## Data Availability

The anonymised data-sets analysed during the current study are not publicly available, but data are available from the corresponding author upon reasonable request.

## Supplementary material

The supplementary material is available online at https://doi.org/10.1192/bjo.2025.56
