# Supplementary material for: Quality of discharge letters concerning readmissions to mental healthcare: case-control study
Source: BJPsych Open. 2025 May 23;11(3):e113. doi: 10.1192/bjo.2025.56 (PMC12188235; doi:10.1192/bjo.2025.56)
Supplement: Hartveit et al. supplementary material [file S2056472425000560sup001.docx]

**Supplementary table 1** Descriptive statistics of patients and discharge letters in the recontact versus no recontact group

|  | Recontact (N=50) | No recontact (N=50) |
| --- | --- | --- |
| Patient sex | Male: 19  Female: 31 | Male: 22  Female: 28 |
| Patient age | Under 25 years: 16  25-45 years: 18  46-65 years: 12  More than 65 years: 4 | Under 25 years: 9  25-45 years: 21  46-65 years: 14  More than 65 years: 6 |
| Received specialised mental health care within the last five years* | Yes: 44  No: 6 | Yes: 28  No: 22 |
| Length of stay/duration of current treatment contact* | < 2 weeks: 27  2 weeks – 6 months: 20  > 6 months: 3 | <2 weeks: 23  2 weeks – 6 months: 15  >6 months: 12 |
|  |  |  |
| Patient main diagnosis groups | Substance abuse: 9 (18%)  Psychosis: 8 (16%)  Bipolar disorder: 6 (12%)  Depression: 4 (8%)  Anxiety, OCD: 5 (10%)  Personality disorder: 9 (18%)  ADHD: 3 (6%)  Adjustment disorders: 4 (8%)  Other: 2 (4%) | Substance abuse: 6 (12%)  Psychosis: 5 (10%)  Bipolar disorder: 5 (10%)  Depression: 10 (20%)  Anxiety, OCD: 7 (14%)  Personality disorder: 1 (2%)  ADHD: 4 (8%)  Adjustment disorders: 5 (10%)  Other: 7 (14%) |
| Number of information items reported in discharge letters^Δ^ | 14.7 (SD=2.9), range 9-20 | 14.3 (SD=2.6), range 7-20 |

**p* ≤ 0.05, *χ2*-test.

^Δ^Information items assessed by the 26 QDis-MH checklist items. OCD: Obsessive-Compulsive Disorder. ADHD= Attention Deficit Hyperactivity Disorder.
